# Construction of higher-order cellular microstructures by a self-wrapping co-culture strategy using a redox-responsive hydrogel

**DOI:** 10.1038/s41598-020-63362-4

**Published:** 2020-04-21

**Authors:** Wahyu Ramadhan, Genki Kagawa, Kousuke Moriyama, Rie Wakabayashi, Kosuke Minamihata, Masahiro Goto, Noriho Kamiya

**Affiliations:** 1 Department of Applied Chemistry, Graduate School of Engineering, Kyushu University, 744 Moto-oka, Fukuoka, 819-0395 Japan; 2 Department of Chemical and Biological Engineering, National Institute of Technology, Sasebo College, Okishin-cho, Sasebo, Nagasaki 857-1193 Japan; 3 Center for Future Chemistry, Kyushu University, Fukuoka, 819-0395 Japan

**Keywords:** Chemical engineering, Cell proliferation, Cancer microenvironment, Biomaterials - cells, Cell biology

## Abstract

In this report, a strategy for constructing three-dimensional (3D) cellular architectures comprising viable cells is presented. The strategy uses a redox-responsive hydrogel that degrades under mild reductive conditions, and a confluent monolayer of cells (i.e., cell sheet) cultured on the hydrogel surface peels off and self-folds to wrap other cells. As a proof-of-concept, the self-folding of fibroblast cell sheet was triggered by immersion in aqueous cysteine, and this folding process was controlled by the cysteine concentration. Such folding enabled the wrapping of human hepatocellular carcinoma (HepG2) spheroids, human umbilical vein endothelial cells and collagen beads, and this process improved cell viability, the secretion of metabolites and the proliferation rate of the HepG2 cells when compared with a two-dimensional culture under the same conditions. A key concept of this study is the ability to interact with other neighbouring cells, providing a new, simple and fast method to generate higher-order cellular aggregates wherein different types of cellular components are added. We designated the method of using a cell sheet to wrap another cellular aggregate the ‘cellular Furoshiki’. The simple self-wrapping Furoshiki technique provides an alternative approach to co-culture cells by microplate-based systems, especially for constructing heterogeneous 3D cellular microstructures.

## Introduction

Recreation of the three-dimensional (3D) architecture of viable cells is an emerging technology^1,2^ for developing tissue-like structures with functions in the field of tissue engineering and as a new cell-based tool in the early phase of drug discovery^3,4^. These bottom-up approaches^5–7^ have attracted significant attention for use in the fabrication of 3D cellular microstructures, including the cell sheet^8^, and multi-cellular aggregate technologies such as microstructure blocks^6^, fibers^7^, spheroids^9^ and organoids^10^. However, the construction of a fully viable, heterogeneous tissue-like structure using 3D cell culture techniques has yet been challenging^11–13^. Thus, it remains a major challenge to establish efficient and effective ways to upgrade recent technologies for 3D cell culture techniques^14–16^, where co-culturing of different types of cells is a promising approach to formulate and to better mimic a natural tissue with a complex heterogeneous 3D cellular microstructures. Additionally, development of a 3D co-culture approach holds great potential for fundamental research efforts and its application toward monoculture systems^2^. Current combinatorial methods for the development of 3D co-culture systems are mainly classified by scaffold-free or scaffold-based techniques^2,17^. Form the viewpoint of cell-to-cell interaction, the co-culture techniques can be categorized by cell contact orders^2^ such as simple and randomly mixed co-culture, segregated co-culture (culturing cells in different plates), sandwich or layered co-culture, cell patterning approach using a designed platform, and cell encapsulation techniques^18^.

Although scaffold-free co-culture techniques have shown a great progress in terms of viability and functionality of various cell sources, from the engineering perspective it is hard to attain the spatial distribution and organization of cells in a 3D cellular microstructure. Conversely, scaffold-based techniques such as transwell plate, bioreactor, microfluid technology, micropatterning and other methods^19–22^. require a specialized method, require more resources, is labour-intensive, and require costly medical procedures^23–25^. In particular, it requires specialized equipment that may not be readily accessible to a standard laboratory. From this context, a cost-effective alternative approach for the construction of 3D cellular microstructures with standard equipment should be of great interest from basic studies to practical applications in biomedical fields^2^.

To date, the configuration of encapsulation-based co-culture system that enables covering cells with another cell prevised a promising technique to increase the cell-to-cell contact during culturing of multiple cells. These systems have been used to evaluate the cellular movement on continual regulation and the response to the physiologic stimuli, e.g., cell invasion, migration, angiogenesis and metastasis in complex tissues. Since the pioneering work of cell encapsulation with another cell coating^26^, cells are often encapsulated within biomaterial-based scaffolds. However, the incomplete adherence of cells on the culture substrate and the difficulties to control the cell density and cellular movement on the outer membrane layer of encapsulated cell have been reported in this system^19,20^. One simple solution to overcome this limitation is to encapsulate target cells directly with an established cell layer from other origins without the aid of scaffold materials.

Cell sheet engineering itself is another powerful approach to co-culture cells^21–23^. Cell sheets are thin confluent monolayers of cells connected to each other in a flat, sheet-like manner^24^, and overlaying the cell sheets enables the construction of heterogeneous 3D cellular structures^25^. In the field of tissue engineering, this system also has been used to graft a cell sheet onto an organ surface, which attenuates deleterious host immune responses toward encapsulated cells used in autologous cell therapy applications^27–29^.

**Figure 1:**
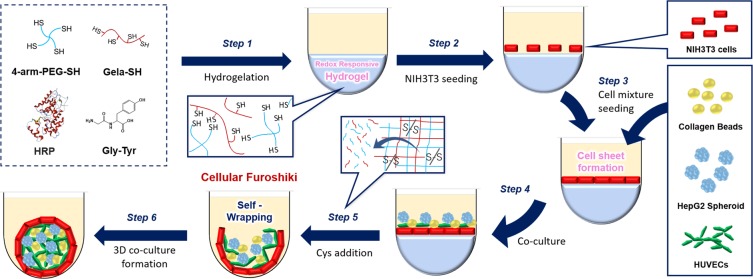
Overall schematic illustration for the fabrication of the ‘cellular Furoshiki’ using redox responsive hydrogels. Step 1: Enzymatic preparation of a redox responsive hydrogel. Step 2: Seeding of NIH3T3 cells on the hydrogel. Step 3: Seeding of collagen beads, HepG2 and HUVECs. Step 4: The cells adhere to the cell sheet surface. Step 5: Hydrogel degradation. Step 6: Self-wrapping of the co-cultured cells. The artwork was designed using Office 365 ProPlus - Microsoft Office PowerPoint (Provided by Kyushu University, version 2016 for Macintosh, Microsoft Corp., Redmond, WA).

The advance of current manipulation methods of living cells motivated us to propose a new way to encapsulate living cells within a confluent monolayer of cells (i.e., cell sheet). To the best of our knowledge, there has been no report on the utilization of a live cell sheet as a foldable cell layer to initiate the co-culture process. Herein, we demonstrate a facile method using a two-dimensional (2D) cell sheet to wrap 3D cellular aggregates and other biological entities. As a proof-of-concept study, a redox-degradable PEG-based hydrogel linked by disulfide bonds^30–33^ that degrades under mild, cell-friendly reductive conditions was used. We found that by simply altering the concentration of cysteine (Cys) the degradation of the redox-responsive hydrogel can be controlled, indicating that detachment of the cell sheet can be regulated. In the present study, we have optimized the self-folding process of a 2D fibroblast (NIH3T3) cell sheet to wrap 3D HepG2 spheroids and other cells (human umbilical vein endothelial cells (HUVECs)) and/or collagen beads into higher-order cellular microstructures (Fig. 1). The aggregated human hepatocellular carcinoma (HepG2) spheroids were employed as a model of small tissue. Increase in the hepatic function of co-cultured HepG2 cells affords opportunities to create a unique microenvironment for multicellular aggregates to promote direct cell-cell contacts, which will benefit further development of a simple, microplate-based co-culture technology. We called the new self-wrapping co-culture strategy ‘cellular Furoshiki’, in which, a cell sheet is used to wrap other cellular aggregates, like the traditional Japanese fabric Furoshiki.

## Results

### Kinetic analysis of the detachment of a cell sheet from the redox-responsive hydrogel

The shrinking ability of the cell sheet is mediated by the interplay between the wrapping behaviour of the cell sheet by redox responsive degradation and the state of other entities including cells on the cell sheet. Initially, NIH3T3 cells were cultured on the redox-responsive hydrogel for 3 d to fabricate the cell sheet (Fig. S1A). Because the rate of degradation of the redox-responsive hydrogel is affected by the reductant concentration, the folding behaviour of the cell sheet detachment from the hydrogel was evaluated by varying the Cys concentration (1–50 mM). In addition, the time required for the complete degradation of hydrogels was assessed.

**Figure 2:**
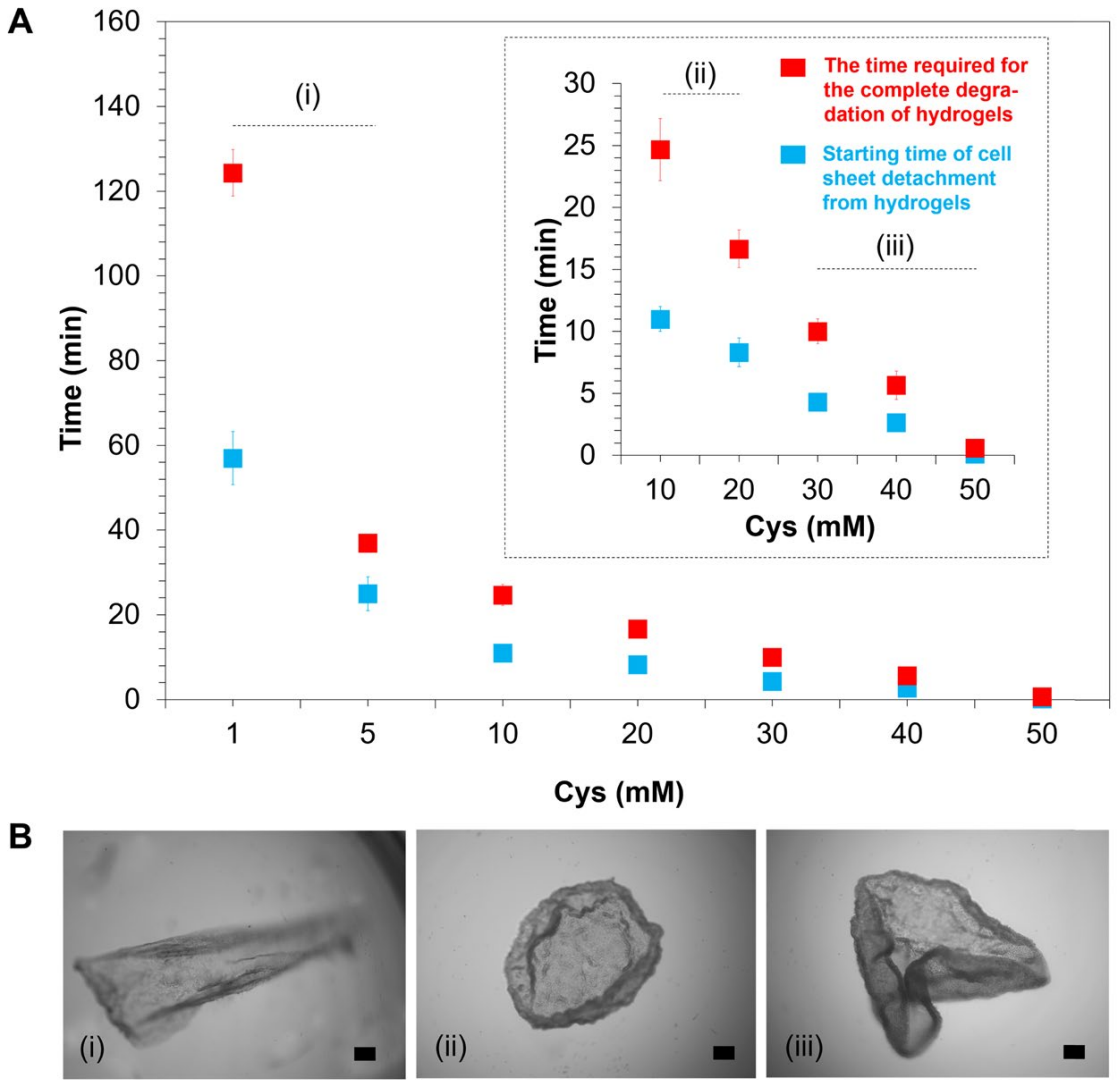
Kinetic behaviour of the detachment of a cell sheet from the redox-responsive hydrogel. (**A**) Effect of cysteine (Cys) concentration on the duration time of cell sheet detachment from the redox responsive hydrogel (*n* = 3). Inset: enlarged figure after 30 min in the presence of 10–50 mM Cys. (**B**) Observation of cell sheet detachment behaviour. Hydrogels were degraded using 1–50 mM Cys. Photo (i), (ii) and (iii) present images of the cell sheet wrapping behaviour after adding 1–5, 10–20 and 30–50 mM Cys, respectively. All photos were captured by Keyence BZ-9000 microscope. Scale bar is 200 µm.

The results showed that the initial time point for detachment of the cell sheet decreased as the concentration of Cys increased (Fig. 2A), whereas the required time for the complete degradation of the hydrogel increased as the Cys concentration decreased. At the highest Cys concentration (50 mM), the cell sheet detached immediately from the hydrogel upon transition to the sol state (within 1 min). In contrast, at the lowest Cys concentration (1 mM), the cell sheet started to fold at 57 ± 6 min, and had completely degraded after incubation for 124 ± 6 min.

The folding behaviour of the cell sheet was observed to gain further insights into the effects of the Cys concentration on cell sheet detachment. Using 1 and 5 mM Cys, cells detached from the hydrogel under mild conditions; however, the detachment only occurred from one side of the cell edge (Fig. 2B-i, Fig. S1B, Vid. S1). Interestingly, in the presence of 10 and 20 mM Cys, detachment occurred from the outer edge of the cell sheet, as expected (Fig. 2B-ii). Further increases in the Cys concentration to 30, 40 and 50 mM dramatically reduced the starting time of cell sheet detachment (less than 1 min); however, the folding process was difficult to control (Fig. 2B-iii). These results demonstrated that solely adjusting the Cys concentration without disrupting the cell-to-cell connection could control the detachment and shrinkage behaviour of the NIH3T3 cell sheet layer. Twenty millimolar Cys was selected for subsequent experiments because gentle cell sheet detachment behaviour was observed at this Cys concentration.

### Self-wrapping behaviour of the cell sheet upon detachment from the redox-responsive hydrogel

**Figure 3:**
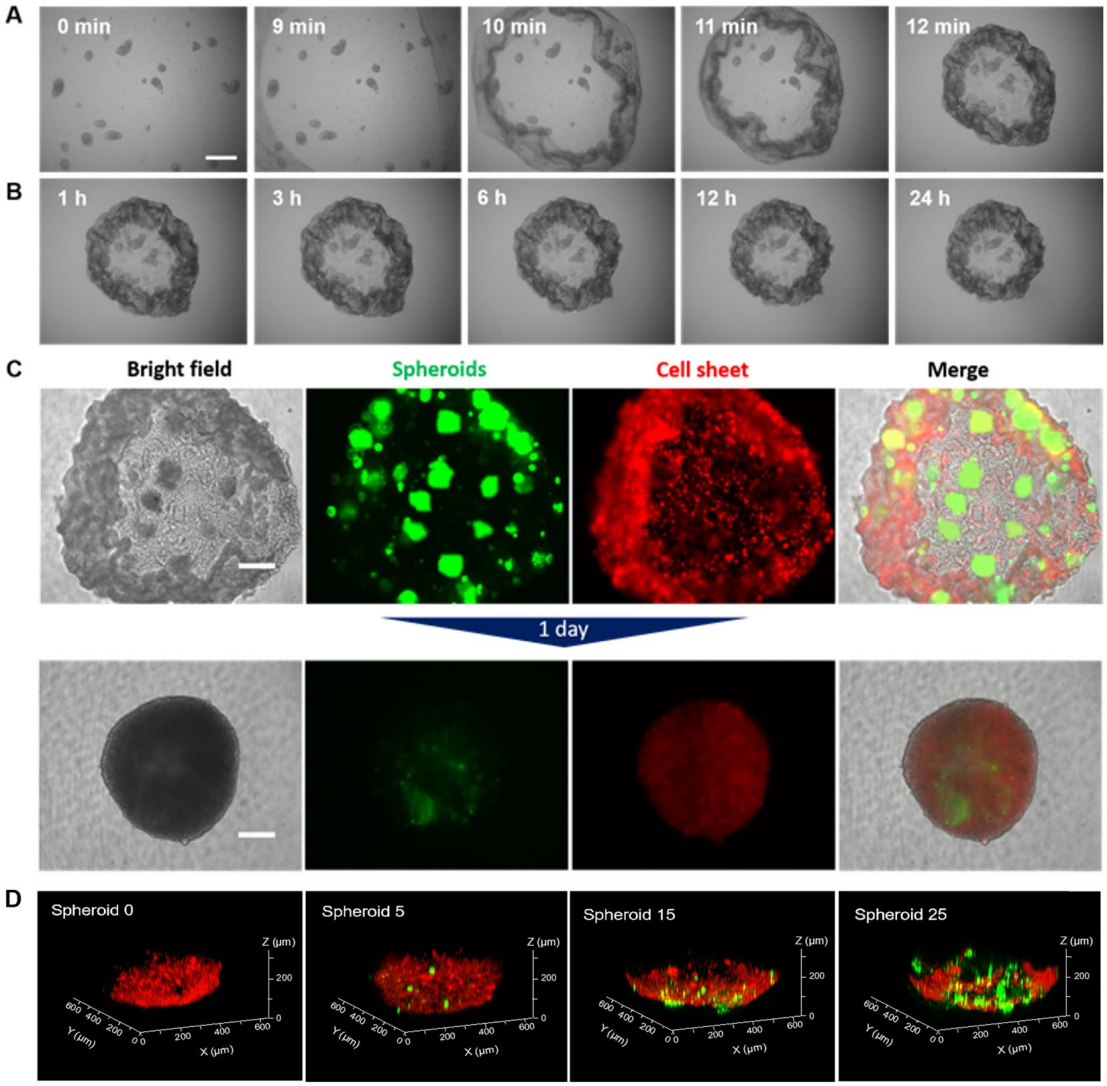
Wrapping spheroids with a cell sheet by the ‘cellular Furoshiki’ technique. (**A**) Phase contrast time lapse imaging of HepG2 cells wrapped by a NIH3T3 cell sheet during the initial folding process (Vid. S2). (**B**) Representative images of the wrapped cellular structure at and after 1 h incubation. The scale bars of A and B are 500 µm. (**C**) Co-existence of HepG2 spheroids in the NIH3T3 cell sheet. Images present the double staining wrapping process between 30 min and 1 d incubation. Images in the right column are merged using the image analysis software BZ Analyzer from the Keyence BZ-9000 microscope. The scale bar is 200 µm. (**D**) Different numbers of HepG2 spheroids in the NIH3T3 cell sheet after 1 d co-culturing. 3D co-culture images were captured by the CLSM-700. The NIH3T3 cell sheet is stained with DiD red fluorescence and HepG2 spheroids are stained with Calcein-AM green fluorescence.

Hepatocellular carcinoma spheroids (HepG2) were immobilize on the cell sheet surface to test the possibility of wrapping other cells on the cell sheet layer during the folding process. Initially, HepG2 spheroids were fabricated by using Elplasia at a specific density (Fig. S2). The harvested spheroids (119 ± 21 µm in diameter) were then co-cultured on the cell sheet (6.5 ± 0.1 mm in diameter) for 4 h. Twenty millimolar Cys was added to the hydrogel and time-lapse observations of wrapping cells was conducted (Fig. 3). As expected, the spontaneous shrinking of the cell sheet began within 10 min after exposure to the aqueous Cys solution (Fig. 3A). During shrinkage, HepG2 spheroids remained attached to the cell sheet. The edge of cell sheets started to fold around 9 min and all HepG2 spheroids were carried to the centre of the well during the wrapping process (Vid. S2). No fragmentation of the cell layer was observed, indicating that the NIH3T3 cell sheet could shrink and hold the spheroids while maintaining the wrapped structure. The visible space between spheroids on the cell sheet decreased with the self-folding process of the NIH3T3 cell layer. After wrapping the spheroids within ~9 min, the top part of wrapped cell aggregates showed an opened structure. Despite no significant change in the whole wrapped structure was observed, the top part of wrapped structure tended to fold gradually in 24 h culture (Fig. 3B) and closed completely at 7 d of co-culture (Fig. S3). Importantly, the coexistence of the spheroids in the inner part of wrapped structure was attained after 1 d of culturing (Fig. 3C).

Various numbers of spheroids (0, 25, 50 and 100 per well) were seeded on the cell sheet to quantitatively analyse the number of spheroids that can be wrapped by the cell sheet (Fig. S4). A large number of spheroids significantly affected the size of the wrapped structure and interfered with the process of cell layering. This condition also affected the closed or opened structure on the top of the wrapped structure during the incubation period. Incorporation of approximately 100 spheroids increased the size of the wrapped structure to more than ~1500 µm. Hence, we reduced the spheroid number to less than 25 per well to maintain the size of the wrapped structure and increase the possibility of incorporating another cell type into the remaining space in the wrapped structure (Fig. 3D). As shown in Fig. 3D, after 1 d incubation without HepG2 spheroids, the cell sheet formed a cellular aggregate with the size ca. ~500 µm. Similar results were observed when five spheroids were loaded into the cell sheet. Further increases in the spheroid number, that is, 15–25 spheroids per well, showed a small increase in the size (i.e., ca. ~ 600 µm) of the wrapped structure.

### Viability of co-cultured cells in the wrapped cellular structure

**Figure 4:**
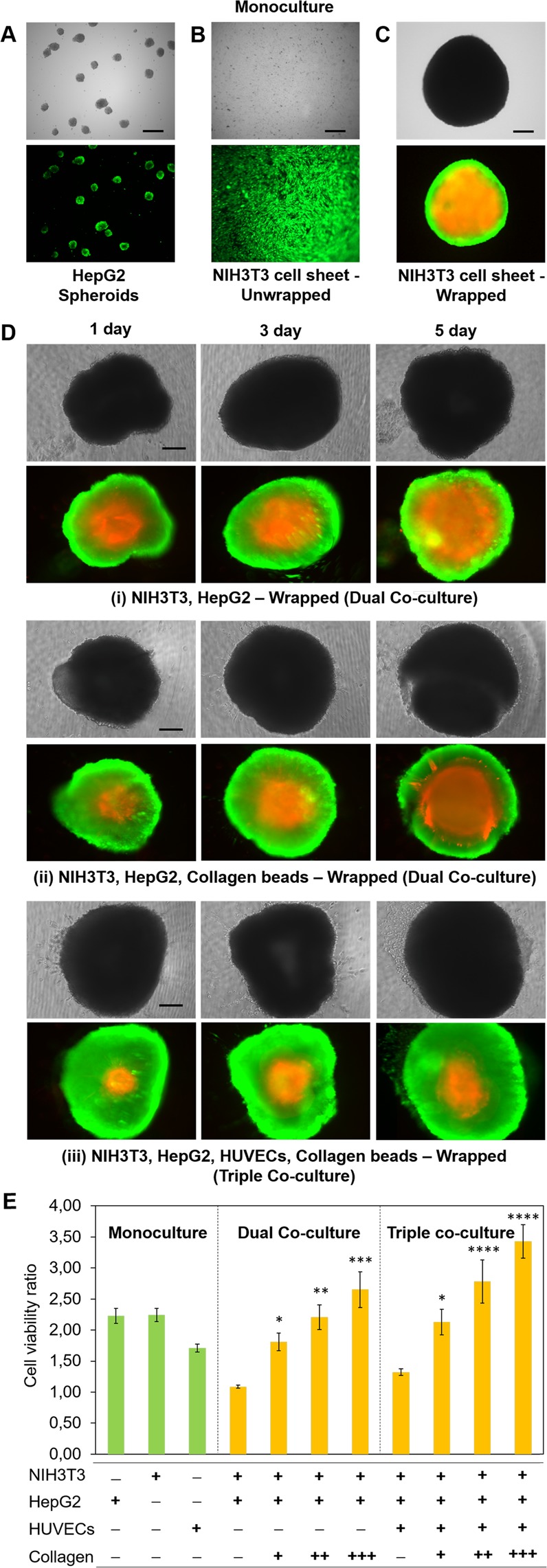
Viability of co-cultured cells in the wrapped cellular structure. (**A**) Cell viability of HepG2 spheroids, (**B**) the NIH3T3 cell sheet and (**C**) the NIH3T3 cell sheet in the wrapped structure state after 5 d culturing. (**D**) NIH3T3-HepG2 co-culturing in the wrapped structure after 1, 3 and 5 d culturing. The initial cell numbers are 15 HepG2 spheroids, 100,000 NIH3T3 cells forming a monolayer and 50 collagen beads. Viable cells are stained green with calcein-AM and dead cells are stained red by propidium iodide. Images were merged directly using the image analysis software BZ Analyzer from the Keyence BZ-9000 microscope. Scale bar is 100 µm. (**E**) Evaluation of the cell viability ratio for different wrapped structures after 5 d culturing. Data of viable cells at day 5 were normalized to cell viability at day 1. The initial cell numbers are 15 HepG2 spheroids, 100,000 NIH3T3 cells forming a monolayer and 4000 cells/well HUVECs. Collagen microparticle numbers are approximately 50 (+), 150 (++) and 250 (+++) in the wrapped cellular structures. Error bars denote standard deviation (*n* = 3). *p < 0.05, **p < 0.01, ***p < 0.001 and ****p < 0.0001 when compared with that of the wrapped structure without collagen beads.

The evaluation of the viability of encapsulated cells in the wrapping network was conducted because a co-culture system exhibits increasing complexity. In this part, we introduced HUVECs (4000 cells/well) and collagen microparticles (50 beads) into the wrapped structure (Vid. S3). The results of the live/dead assay showed that after 5 d culturing only a few dead cells were observed in the HepG2 spheroid (Fig. 4A) and NIH3T3 cell sheet (Fig. 4B). However, incubation for an additional day gave a larger number of dead cells in the monoculture (Fig. 4C) and dual co-culture (Fig. 4D-i) in the centre of the wrapped structure. Comparable results were found with HepG2 spheroids and collagen beads inside the network (Fig. 4D-ii). A significant increase of the necrotic zone and decrease of the quiescent zone were observed after incubating the wrapped structure for 3 and 5 d. During the dual co-culture, the necrotic zone (the red fluorescent region) increased from 48% (1 d) to 87% (5 d) in the total area of the wrapped structure (Fig. 4D-i), while with the inclusion of collagen beads inside the wrapped structure, the necrotic zones were reduced from 34% to 59% for 1 and 5 d culturing, respectively (Fig. 4D-ii). Interestingly, incorporating HUVECs on the cell sheet persevered cell viability. Despite the region of the necrotic zone slightly increased from 24% (1 d) to 41% (5 d), the relative portion of dead cells of the wrapped structure clearly reduced when compared with those of the wrapped structures without HUVECs (Fig. 4D-iii). Accordingly, after 5 d culturing, the results of cell viability with the wrapped co-culture system increased significantly, especially when raising the number of collagen beads in dual and triple co-culture conditions (Figs. 4E and S5).

### Metabolism of co-cultured cells inside the wrapped cellular structure

**Figure 5:**
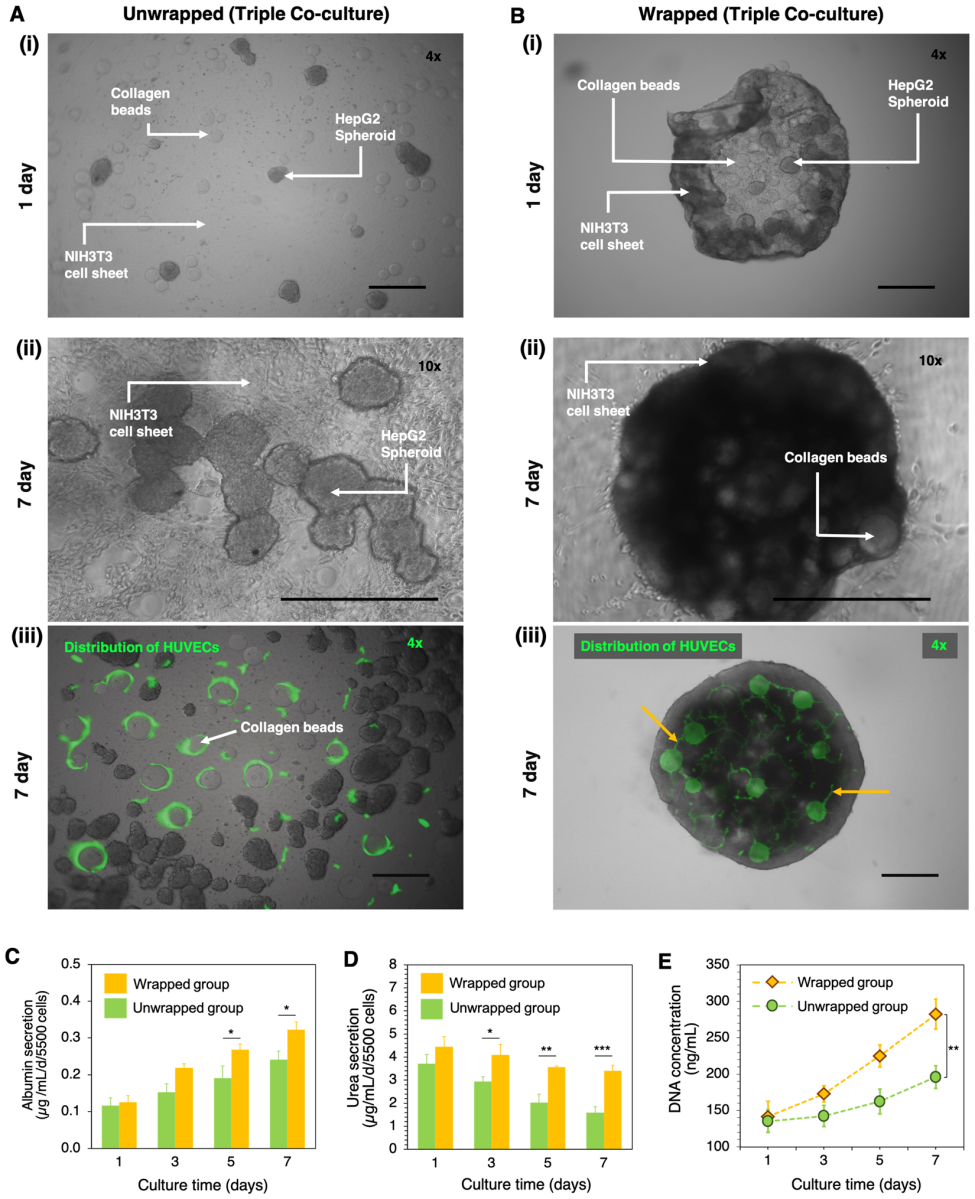
Comparison of metabolism of co-cultured cells between the unwrapped and wrapped cellular structures. Unwrapped (**A**) and wrapped (**B**) groups of triple co-cultured HepG2, HUVECs and collagen beads on the NIH3T3 cell sheet. Representative phase contrast images of unwrapped group (A-i) and wrapped group (B-i) after 1 d of co-culture (images were taken at 4X magnification). Representative phase contrast images of unwrapped group (A-ii) and wrapped group (B-ii) after 7 d of co-culture (images were taken at 10X magnification). The existence of NIH3T3, HepG2 spheroid, and collagen beads are indicated by white arrows and labelling. Representative images of CD31 staining (green fluorescence) of HUVECs on the unwrapped structure (A-iii) and within the wrapped structure (B-iii) (images were taken at 4X magnification). A connected part of HUVECs is indicated by orange arrows. The co-culture images were captured by CLSM-700. Scale bar is 500 μm. Albumin (**C**) and urea (**D**) secretion from wrapped and unwrapped triple co-cultured cells for 7 d. (**E**) Quantitative data for proliferation of the triple co-cultured cells as determined by DNA content. Error bars denote standard deviation (*n* = 3) and *p < 0.05, **p < 0.01, ***p < 0.001.

To assess the function of HepG2 spheroids under triple co-culture conditions inside the wrapped structure, we evaluated metabolism from the co-culture cells in unwrapped and wrapped groups. The triple co-culture cells consisted of 15 spheroids of HepG2, 4000 cells of HUVECs and 50 collagen beads on the confluent of NIH3T3 cell sheet. In the unwrapped group, cells were cultured on the hydrogel without the degradation process (2D culture) (Fig. 5A-i,A-ii), whereas in the wrapped group cells were adhered onto the NIH3T3 cell sheet and were wrapped and packed after the addition of 20 mM Cys (Fig. 5B-i,B-ii)). HUVECs were randomly attached to the surface of collagen beads in the unwrapped structure (Fig. 5A-iii), in contrast in the wrapped structure the beads were fully covered with adhered HUVECs, which results in the formation of a capillary-like structure under the culture period (Fig. 5B-iii). We examined the effect of the two different culturing conditions by measuring the secretion of albumin, urea and DNA contents for 7 d of culturing (Fig. 5C–E). We observed that the secretion of albumin in both unwrapped and wrapped groups increased as the culturing period increased with the secretion level in the wrapped group significantly higher (*p < 0.05) than that in the unwrapped group after 5 and 7 d of culturing (Fig. 5C). Urea secretion decreased during the culturing period for both groups with a significant difference (***p < 0.001) in urea secretion between the unwrapped and wrapped groups observed after 7 d incubation with values of 1.6 and 3.4 µg/mL/d/5500 cells, respectively (Fig. 5D). The quantitative data of cellular proliferation within co-cultured HepG2 spheroids and HUVECs on the NIH3T3 cell sheet were examined by measuring DNA content. DNA synthesis was observed to increase for both groups over the 7 d culturing period with the wrapped group showing higher levels of cell proliferation (Fig. 5E). At 7 d, the wrapped group had a 38.4% greater level of DNA synthesis when compared with the unwrapped group (**p < 0.01).

## Discussion

Most studies have explored how to harvest intact cell sheets from scaffolds^28,34^. In contrast, there are only a few studies that have attempted to fold a cell sheet by cells detaching from the substrate, especially to manipulate the wrapping-process to form 3D or higher-order cellular microstructures by co-culturing with other cells. In the previous reports showing the immune response of transplanted tissues layered with a cell sheet^26,35,36^, a cell scraper had been used to harvest the cell sheet, which may weaken the cell junctions and ECM condition. It is well reported that in the harvesting step, cell sheet needs a supporting material and manipulation technique to prevent the shrinking and folding of cytoskeleton during the detachment processes^34^. Eventually, the harvesting process of cell sheets is hard to control in general, thus it was not feasible to stratify the cell sheet to the other cells, spheroids, or small tissue.

Inspired by previous encapsulation technologies of cells, herein we proposed a self-wrapping technique with a cell sheet. Fibroblast cell sheet was utilized as a model of confluent monolayer cells to wrap the immortal liver cells (HepG2 spheroids). The underlying idea behind this work is the cell-cell interaction theory where encapsulation of living cells with other cells should extend cellular functions^37^. When the cell sheet detaches from the basement of the culture substrate, a contractile force produced by actin filaments pulls the neighbouring cells and 3D spheroids on the cell sheet, which leads to the wrapping process, that is, a core-shell type higher-order cellular aggregate is obtained. We postulate that this design and construction of a co-culture system by the wrapping cell sheet process should provide an alternative cell culture system. We used a stimuli-responsive cell culture substrate based on polymeric materials to achieve this goal^38–40^.

The redox-responsive hydrogel system is a promising stimuli-responsive matrix that has been used to fabricate live fibroblast cell sheets^31,41,42^. Biological entities cultured on the disulfide-linked PEG-based hydrogel were harvested by cleaving the S–S bonds in the polymeric network. Cysteine was selected because it is a mild reductant under physiological conditions when compared with that of glutathione, β-mercaptoethanol, dithiothreitol and other reducing agents^31,43,44^. We showed previously that the redox-responsive hydrogel degradation rate and complete detachment of the cell sheet typically occur ~30 min after incubation with 5 mM Cys^31^. However, the effect of the Cys concentration on harvesting, specifically the folding process of the fibroblast cell sheet, has not been examined. Therefore, the kinetic behaviour of cell sheet detachment from the hydrogel by varying the Cys concentration was examined. The folding process of the cell sheet was systematically controlled, and the hydrogel was readily degraded with 20 mM Cys under cyto-compatible conditions. Our results showed that spontaneous shrinkage began within 9 min after exposure to Cys, and all HepG2 spheroids were dispatched successfully to the centre of the well. The presence of HepG2 spheroids on the cell sheet did not affect the cell detachment behaviour but affected the size and structure (opened or closed on the top) of the resultant wrapped structure and the viability of the co-cultured cells significantly. Thus, controlling the spheroid number was critical for effective cellular wrapping, and the HepG2 cell function in the spheroid form was expected to increase heterogeneous cell-cell interactions. Here, we selected ~15 spheroids to ensure that the size of the wrapped structure did not increase dramatically and that bare areas were available for the incorporation of another cell type.

The viability ratio of the HepG2 spheroids in the wrapped structure was significantly lower when compared with that of HepG2 cells in monoculture (Fig. 4A). The hypoxic areas found in the central part of the wrapped structure are caused by insufficient permeation of oxygen because of the thickness of the cell sheet structure. Although the top of wrapped cell aggregates opened to bulk culture medium, the necrotic area in the multicellular spheroid was a predictable condition and is a general problem in the construction of 3D cell cultures^45^. According to previous report, the size limit of normal engineered tissue is around 100–200 μm^46,47^ because of the inadequate gas exchange, nutrients, and elimination of cellular waste product^48^. We found that gradual increase in the size of wrapped structure (initially ~ 500 μm) by 7% (Fig. 4D-i), 13% (Fig. 4D-ii) and 16% (Fig. 4D-iii) after in 5 d culturing by introducing collagen beads and HUVECs.

Collagen is a major component of the extracellular matrix (ECM) and plays an important role in artificial scaffold developments for the alignment and organization of cells^49^. Various types of collagen microparticles have been used for *in vitro* 3D cell culture engineering^16,50–52^. We thus produced collagen (type I) beads^50^ and placed them on the cell sheet before the cell sheet detachment. Inclusion of collagen beads into the NIH3T3 cell sheet and HepG2 spheroids increased the viability of the co-cultured cells. The necrotic area was reduced from 87% (without collagen beads) to 59% (with collagen beads) of the total area of the wrapped structure (Fig. 4D-i,D-ii).

However, designing large 3D cellular microstructures while maintaining cell viability still remains a challenge. Based on the ability to distribute oxygen, metabolites and nutrient, presenting endothelial cells are favorable in tissue engineering fileld^48^, especially for promoting vascularization in the 3D cell culture^53–55^. The incorporation of endothelial cells (e.g., HUVECs) was thus used in an attempt to improve the cellular function of the wrapped structure (triple co-culture, Fig. 4D-iii)^53,56^. Interestingly, incorporating HUVECs improved the viability of co-culture cells when compared with the other wrapped structures without HUVECs (Fig. 4D). This is possibly because HUVECs provide a crucial role in regulating interactions between cells by forming microvascular structures^53,57^. In the presence of HUVECs, the cell viability rate also increased when collagen beads were included (Fig. 4E); however, this increase differed from the co-cultured group without HUVECs. These results indicate that cellular interactions between HepG2 and HUVECs achieved a superior performance when compared with just HepG2 and NIH3T3 cells^58,59^. Although NIH3T3 cells have been shown to support hepatocytes in maintaining their differential function for long periods, NIH3T3 cells or fibroblasts are not in physical contact with hepatocytes in native liver tissue^59,60^. Naturally, hepatocytes and HUVECs together account for more than 80% of the liver of mass^61^. Importantly, the wrapped cellular structure of HepG2, HUVECs, and collagen beads inside the NIH3T3 cell sheet (triple co-culture with collagen beads) gave significantly higher cell viability than HepG2 spheroids alone, indicating that this self-wrapping technique is capable of maintaining healthy conditions for co-culturing cells by appropriate combinations of different cell types.

Since the increasing number of collagen beads either in dual or triple co-culture conditions gave significant effect to the cell viability of the wrapped structure, collagen beads might work as a spacer and concurrently as a scaffold in the wrapped structure. Yamada and coworker^50,62^ reported that collagen beads have function to create an internal conduit space for the effective diffusion of nutrients and oxygen to the center of the cellular aggregates. The increasing of the cell viability is strongly related to the opened structure of wrapped cells, where the increasing in the number of collagen beads results in the larger opened structure of that system (Fig. S5). Organization of collagen beads might facilitates the diffusion of the culture medium to the centre of the wrapped structure^48^. Owing to the adhesive property of collagen type I to enhance cell adhesion on the surface, collagen beads have also played a role as a scaffold to promote the growth of HUVECs. Accordingly, the inclusion of HUVECs could enhance cell-ECM interactions to increase the cell viability (Fig. 4E).

Comparison of the wrapped structure (triple co-culture with collagen beads) with the unwrapped structure was performed for 7 d of culturing. The results showed not only clear differences in morphology, but also significantly improved urea and albumin secretion as the HepG2 specific functions for the wrapped co-culture system (Fig. 5C,D). In the wrapped structure, the HepG2, HUVECs and collagen beads were surrounded by the cell sheet and were packed into a higher-order microstructure. The large contact area among cells provided an increase in cell-to-cell interactions to enhance the higher cellular functions of HepG2. In contrast, in the unwrapped group, HepG2 cells adhered to form spheroidal structures on the surface of HUVECs and NIH3TH cells after 7 d of culturing.

The distribution of HUVECs was clearly visualized by the CD31, a marker protein highly expressed on the endothelial cell membrane^63^ (Fig. 5A-iii, B-iii). Figure 5B-iii shows that HUVECs proliferated around the collagen beads and well-dispersed in the wrapped structure. The adherence behavior and distribution of cells on the collagen beads surface was similar to that found in previous report^16^. Since the positive stain of CD31 relates to the initial step of angiogenesis and migration^64^, HUVECs located in the interspace of collagen beads could form a capillary-like structure^59,65,66^.

In general, hepatocyte (either normal cell or immortal cell) is a cell of the main parenchymal tissue of the liver^58,67^. One of the detoxifying functions is to modify ammonia into urea for excretion. While albumin is often employed as an important secreted protein in the liver metabolism^58^. Both of urea and albumin is generally employed as a marker of hepatocyte metabolic activity *in vitro* and to evaluate the liver-specific function. The wrapped structure showed marked increases in the secretion of albumin (1.3-fold), urea (2.1-fold) and DNA content (1.4-fold) when compared with that of the unwrapped structure. A similar trend were reported in previous works where the presence of hepatocyte in connected culture with endothelial cell gave a positive effect on the urea and albumin synthesis compared with a monoculture system^67–70^. Since DNA content is a measure of cell proliferation^71^, dsDNA was selected as the representative of the proliferation rate of wrapped structures. The increase in metabolism and DNA content indicated a better interplay among the wrapped cells, HepG2, HUVECs and collagen during the co-culturing. It is notable that we can adjust the number of HepG2 spheroid, the amount of collagen beads, and the presence of HUVECs that affects cellular morphology and physiological responsiveness of the resultant wrapped structure.

Finally, ‘Furoshiki’ is recognized as a perfect fabric design for wrapping valuable items. Inspired by the traditional engineered Japanese item, we presented a self-folding NIH3T3 cell sheet that wrapped biological entities and termed this the ‘cellular Furoshiki’ technique. As demonstrated, the cell sheet was capable of wrapping other cells, and thus the cellular Furoshiki should provide an alternative approach for constructing complex, higher-order cellular microstructures. Although the main focus of this study is to investigate the potential of a confluent cell monolayer in a self-wrapping co-culture technique, we envisioned a possible application of the cellular Furoshiki either in the development of *in vitro* disease model for the drug screening application^3,72,73^ and or the tissue engineering for the prevention of the rejection of immunosuppression in transplantation^74–76^.

## Conclusions

We have demonstrated the cellular Furoshiki technique as a new construction technique for the design of a higher-order cellular microstructure composed of a NIH3T3 cell sheet, HepG2 spheroids, HUVECs and collagen beads. Compared with the conventional co-culture system (i.e., unwrapped system), the cellular Furoshiki provided an increase in cell viability and metabolism of cellular components. The present concept is based on a simple microplate-based cell culture technique, which is accessible to standard laboratories. Future challenges include the design of tissue-like structures by integration of the cellular Furoshiki presented herein with other cell lines toward practical applications in biomedical fields.

## Materials and Methods

### Materials

PTE-200 SH (Sunbright) (4arm PEG-((CH_2_)_2_-SH)_4_, Mw 20 kDa) was supplied by the NOF Corporation (Tokyo, Japan). Glycyl-L tyrosine hydrate and 1-ethyl-3-(3 dimethyl aminopropyl) carbodiimide (EDC) were purchased from Tokyo Chemical Industry (Tokyo, Japan). Horseradish peroxidase (HRP; activity 100 unit/mg) was purchased from Wako Pure Chemical Industries (Osaka, Japan). Gelatine type A was purchased from Sigma-Aldrich (St Louis, MO, USA). 5,5′-Dithiobis (2-nitrobenzoic acid) (DTNB) and the Cell Stain-Double Staining kit were purchased from Dojindo (Kumamoto, Japan). L-Cysteine (Cys) was supplied from TCI Chemicals (Tokyo, Japan). Trypan blue (0.4%), Minimum Essential Medium (MEM) (1×) + GlutaMAX-I, 10% fetal bovine serum (FBS) and MEM Non-Essential Amino Acids solution were purchased from Thermo Fisher Scientific (Waltham, MA, USA). Cystamine, 1% antibiotic-antimycotic, trypsin 0.25%/1 mM EDTA and Dulbecco’s phosphate buffer saline (D-PBS) were purchased from Nacalai Tesque (Kyoto, Japan). Endothelial growth medium (EGM-2) in the presence of FBS, hydrocortisone, growth factors (including hFGF, VEGF, R3-IGF-1 and hEGF), ascorbic acid and GA-1000 was supplied by Lonza (Walkersville, MD, USA). Collagen type I (bovine skin), the urea quantification assay kit (DIUR-100, BAS) and the human albumin ELISA quantitation set were purchased from Funakoshi (Tokyo, Japan). The Vybrant DiD Cell-Labeling solution kit was acquired from Biotium (Fremont, CA, USA). Elplasia micro space cell culture plates (MPC 3506) were purchased from Kuraray (Okayama, Japan) and Prime Surface non-adherent 96-well plates MS-9096 U were from Sumitomo Bakelite (Tokyo, Japan). Ultra-high pure water was used during experiments (Milli-Q Integral MT3S.kit, Tokyo, Japan).

### Fabrication of the redox responsive hydrogel

The hydrogel was prepared by HRP-mediated crosslinking of thiolated polymers with a slight modification^31^, and Gela-SH was prepared by following the protocol in our previous study^32^. In brief, a specific amount of 4-arm PEG-SH (5%, w/v), Gela-SH (0.01%, w/v) and Gly-Tyr (5 mM) were dissolved in D-PBS (pH 7.4). Subsequently, an aqueous solution of HRP (5 U/mL) was added to the mixture and mixed immediately by gentle pipetting. The reaction and hydrogelation proceeded by incubation at 37 °C for 4 h.

### Cell lines and cell-culture conditions

The NIH3T3 (RCB1862) and HepG2 (RCB1648) cell line were obtained from the Riken Cell Bank (Tsukuba, Japan) The HUVECs (KE-4109) was purchased from KURABO (Osaka, Japan). All cells were maintained as recommended. Briefly, NIH3T3 were maintained in MEM (1×) + GlutaMAX-I and 10% FBS. HepG2 cells were cultured in MEM supplemented with non-essential amino acids (NEAA) and 10% FBS. All media were further supplemented with 1% antibiotic-antimycotic. HUVECs were maintained in EGM-2. For long-term co-culturing of the cell sheet, HepG2 and HUVECS, the heterogeneous cell mixture was prepared in Dulbecco’s minimum essential media (DMEM, Gibco) and EGM-2 (HUVEC basal medium) at a ratio of 1:1, supplemented with the F-12 nutrient mixture (Gibco) and recommended growth factors. Cells were maintained in a humid atmosphere at 37 °C with 5% CO_2_.

### Preparation of the NIH3T3 cell sheet, HepG2 spheroids, HUVECs and collagen beads

The NIH3T3 cell sheet was prepared by seeding the cells on the redox responsive hydrogel. The redox responsive hydrogel was fabricated in 96-well non-adherent plates (MS-9690U). The total volume of the hydrogel was 20 μL per well. After hydrogelation, 100 μL of MEM was added, which contained NIH3T3 cells (3.4 × 10^4^ cells/mL) and incubated for 3 d. HepG2 spheroid cells were fabricated by using the 6-well plate Elplasia system that has 648 microholes. The HepG2 cells density was 2.4 × 10^4^ cells/mL or 150 cells/microhole. The cells were cultured with MEM-NEAA and the medium was changed on the second day. The addition of HepG2 spheroids to the cell sheet was calculated by diluting the 648 spheroids stock to the targeted spheroid number (0–100 spheroids per well). HUVECs were cultured and maintained in EGM-2 medium for 5 d, followed by subculturing to obtain 4.0 × 10^3^ cells/well. Collagen beads or collagen microparticles were prepared by the membrane emulsification method^50,62^, and the number of beads was counted by using a haemocytometer.

### Observation of the wrapping process and cell viability characterization

The required Cys concentration (1–50 mM) as a reductant was examined by measuring the duration time of hydrogel degradation, the initial time of cell sheet detachment and the folding behaviour of the cell sheet. The required time for complete degradation of the hydrogel was determined by measuring the duration time during transformation from the gel state to the solution state. The start time and lapping images were recorded using a Keyence Microscope BZ-9000 from BIOREVO (Tokyo, japan).

The behaviour of cell sheet detachment in the presence of other cells was evaluated by integrating HepG2 spheroids on the cell sheet. The co-cultured cells were incubated for 4 h at 37 °C with 5% CO_2_ to ensure the spheroids adhered to the cell sheet. After 4 h incubation, the medium was removed and 100 μL Cys solution (20 mM) was added to each well. Observation of the initial wrapping process was conduct just after Cys addition. The initial wrapping process was recorded every 15 min and after 1 h the wrapping process was recorded at 3, 6, 12 and 24 h incubation time points by using the Keyence Microscope. Optimization of the wrapping process was performed in the absence and presence of 5, 15, 25, 50 and 100 spheroids per cell sheet, and observations were conducted using a confocal laser scanning microscope (CLSM) LSM-700 from ZEISS (Tokyo, Japan) after 1 d of culturing.

The imaging of cell viability was performed by double stain Calcein-AM for live cell and propidium iodide red staining for recognition of dead cells. Fifteen HepG2 spheroids, 4.0 × 10^3^ cells/well HUVECs and 50 collagen beads were added onto the cell sheet. The wrapped cellular structure was fabricated by adding 20 mM Cys. Only HepG2 spheroids and only NIH3T3 samples in the wrapped and unwrapped conditions were prepared in wells as controls. The medium was changed every day, and observations were conducted using the CLSM at 1, 3 and 5 d of co-culturing. The collagen bead number was increased to 50, 150 and 250 beads per well, and the cell viability ratio was quantified using a Cell Counting Kit-8 (WST-8; Dojindo Laboratories) according to the manufacturer’s protocol. After incubation for 5 d, the absorbance of WST-8 at 450 nm was measured using a microplate reader. The cell viability ratio was defined as:

1 $${\rm{Cell}}\,{\rm{viability}}\,{\rm{ratio}}=\{{{\rm{Abs}}}_{450}({5}^{{\rm{th}}}\,{\rm{day}}\,{\rm{living}}\,{\rm{cell}})-{{\rm{Abs}}}_{450}({\rm{blank}})\}/\{{{\rm{Abs}}}_{450}({1}^{{\rm{st}}}\,{\rm{day}}\,{\rm{cell}}\,{\rm{number}})-{{\rm{Abs}}}_{450}({\rm{blank}})\}$$

To validate the function of the wrapped cellular structure as a co-culture system, comparison of a wrapped co-culture and unwrapped co-culture were defined by measuring particular metabolic markers for 7 d culturing. The amount of albumin and urea was measured using the Human Albumin ELISA Quantitation Set and QuantiChrom Urea Assay Kit, respectively, and according to the manufacturer’s instructions. To evaluate the DNA content, the collected co-culture cells were suspended in 0.5 mL 0.2% Triton X-100 solution, sonicated in an ice bath and centrifuged at 5000 × *g* for 5 min (4 °C). Then, 20 μL of the cell lysate was diluted with 80 μL Tris-EDTA buffer (pH 9) and incubated with 100 μL of the working solution of dsDNA reagent in 96-well plates for 2–5 min at room temperature. The DNA concentration in the cell lysate was determined using a Quant-iT Picogreen dsDNA assay kit (Invitrogen, Thermo Fisher Scientific). The DNA content of each sample was determined by measuring the florescence intensity of the mixed well with a SpectraMax i3x Multi-Mode microplate reader (Molecular Devices, Osaka, Japan), with excitation at 480 nm and emission at 520 nm. Data were analysed by plotting fluorescence intensity versus DNA concentration.

### Immunofluorescence staining of HUVECs

Direct immunofluorescence staining was conducted to visualize the distribution of HUVECs in the wrapped cellular structure. Briefly, after 7 d co-culture of cell, the wrapped cells were washed three times with PBS, and fixed with 4% paraformaldehyde for 30 min at 4 °C. The samples were then incubated in 0.1% Triton X-100 in PBS for 20 min at room temperature. Wrapped cells then incubated for 1 hour with the mouse anti-human platelet endothelial cell adhesion molecule (PECAM-1) or cluster of differentiation 31 (CD31) antibody conjugate with FITC (eBioscience, clone 390, USA), at a dilution of 1:20 in PBS. After washing with distilled water, the samples of the wrapped cells were then ready for observing using CLSM-700 as above under excitation and emission wavelengths of 490 nm and 530 nm for FITC. The fluorescence signal was merged with the phase contrast image to confirm the position of HUVECs in the wrapped structure.

### Statistical tests

Data were normalized by the Kolmogorov−Smirnov test. Significant differences of monocultures compared with that of dual and triple co-cultured cells were determined using a Tukey’s multiple comparison test following a one-way analysis of variance. The secretion of albumin and urea, and DNA concentration data from wrapped cells and unwrapped cells were compared by t-test analyses. Data analysis was conducted with Graph Pad Prism 6 and the level of significance was set at *p < 0.05, **p < 0.01, ***p < 0.001 and ****p < 0.0001.

## Supplementary information

Supplementary Information

Supplementary Video 1

Supplementary Video 2

Supplementary Video 3
