# Supplementary material for: Construction of higher-order cellular microstructures by a self-wrapping co-culture strategy using a redox-responsive hydrogel
Source: Sci Rep. 2020 Apr 21;10:6710. doi: 10.1038/s41598-020-63362-4 (PMC7174313; doi:10.1038/s41598-020-63362-4)
Supplement: Supplementary file 1 — Supplementary Information [file 41598_2020_63362_MOESM1_ESM.docx]

***Supplementary Information***

**Construction of higher-order cellular microstructure by a self-wrapping co-culture strategy using redox-responsive a hydrogel**

Wahyu Ramadhan^1^, Genki Kagawa^1^, Kousuke Moriyama^2^, Rie Wakabayashi^1^, Kosuke Minamihata^1^, Masahiro Goto^1,3^, Noriho Kamiya^1,3^*

^1^ *Department of Applied Chemistry, Graduate School of Engineering, Kyushu University, 744*

*Motooka, Fukuoka 819-0395, Japan.*

^2^ *Department of Chemical and Biological Engineering, National Institute of Technology, Sasebo College,*

*Okishin-cho, Sasebo, Nagasaki, 857–1193, Japan*

^3^ *Center for Future Chemistry, Kyushu University, Fukuoka 819-0395, Japan*

*Corresponding author. Tel: +81 92 802 2806; Fax: +81 92 802 2810; Address: Department of Applied Chemistry, Graduate School of Engineering, Kyushu University, 744 Motooka, Fukuoka 819-0395, Japan. E-mail address: kamiya.noriho.367@m.kyushu-u.ac.jp, (N. Kamiya).

**Contents**

1. **Experimental Design** 2
2. **Supplementary Figures** 3

2-1. NIH3T3 cells sheet formation using redox responsive hydrogel 3

2-2. HepG2 spheroid formation using Elplasia System 4

2-3. Evaluation of closed structure of wrapped structure during co-culturing 5

2-4. Evaluation of spheroid number in the wrapped structure 6

5-5. Evaluation of collagen beads number in the wrapped structure 7

1. **Supplementary Videos** 8
2. **References** 11

**1. Experimental design**

Overall experimental scheme of this study is shown below.

**
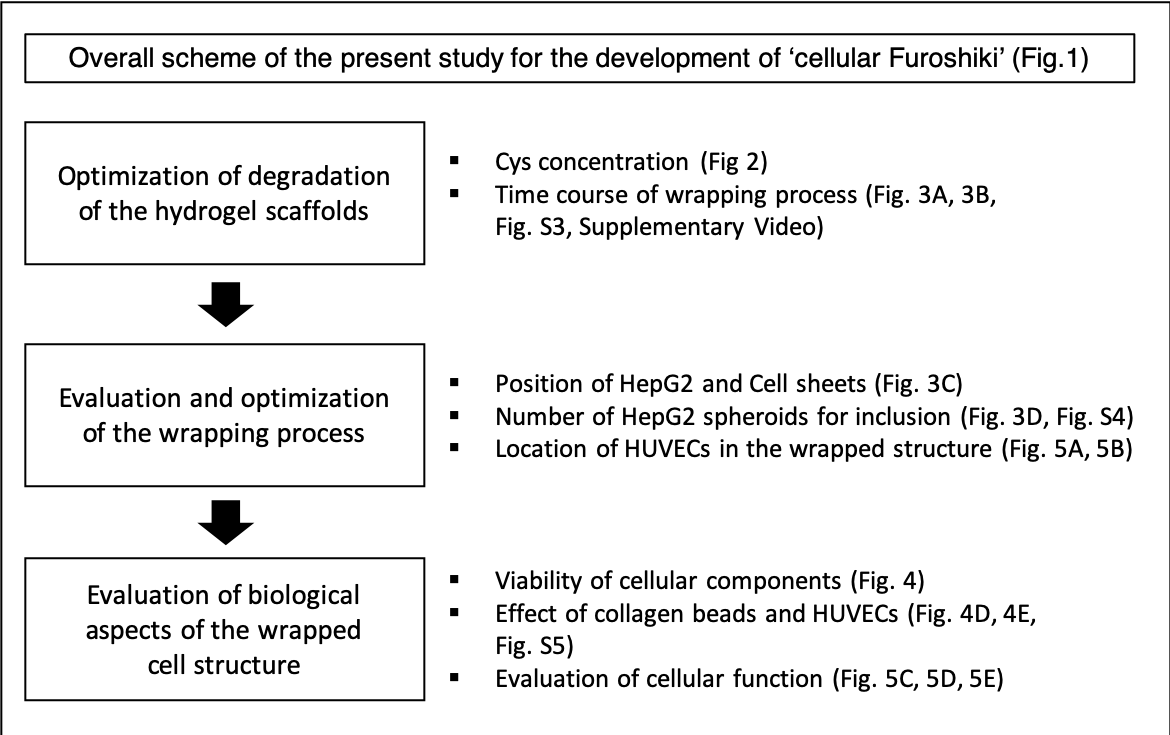
**

**2. Supplementary Figures**

**2-1. NIH3T3 cells sheet formation using redox responsive hydrogel**

Fabrication of the redox responsive hydrogel was adapted from previous studies^1^. Briefly, PEG-SH, Gly-Tyr, Gela-SH, and HRP were dissolved in D-PBS. The concentrations of PEG-SH, Gela-SH, Gly-Tyr, and HRP were 5% (w/v), 0.01% (w/v), 5 mM, and 5 U/mL, respectively. The gels were prepared at a total volume of 20 μL in the non-adherent 96-well plates and incubated for 4 h at 37 °C with 5% CO_2_. After hydrogelation, 100 μL of MEM was added which contained NIH3T3 cells (3.4 x 10^4^ cells/mL) and incubated for 3 d. After confluent, 5 mM Cys was added to the hydrogel system.

**
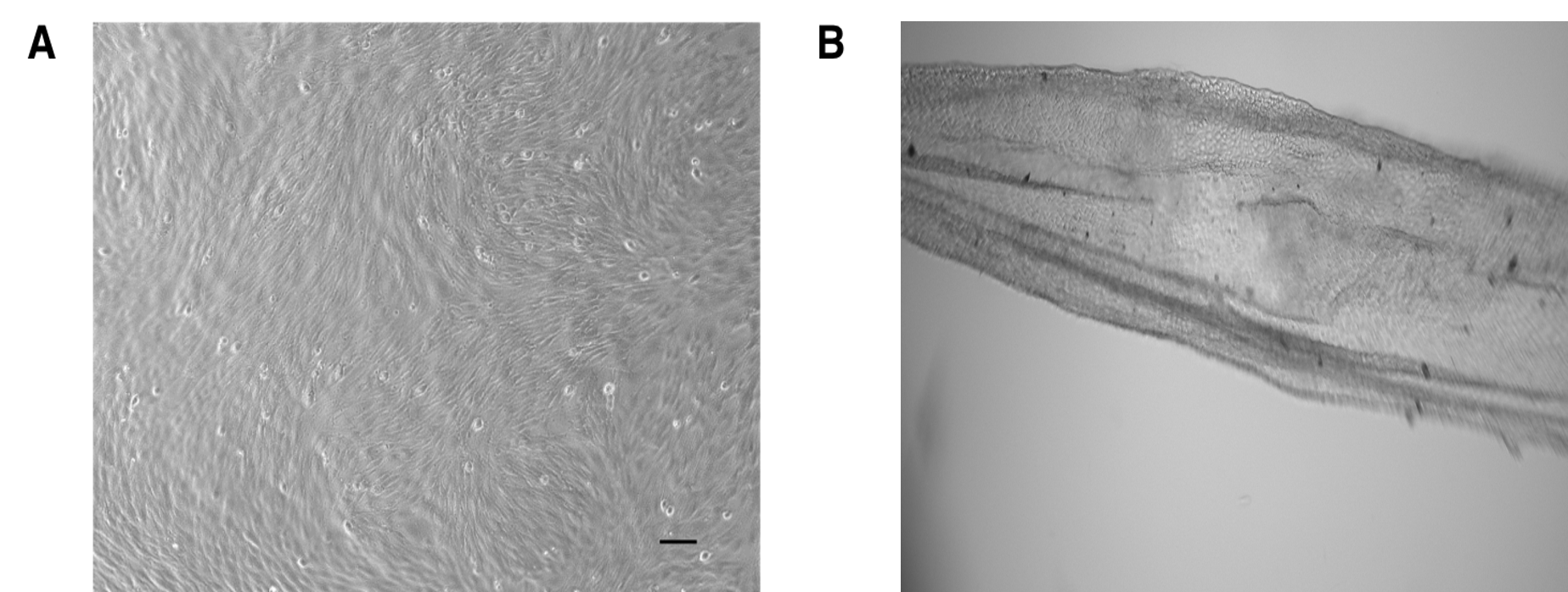
**

**Fig. S1**. A. NIH3T3 Cell sheet formation on the redox responsive hydrogel after 3 d incubation. B. Hydrogel was degraded after the addition of 5 mM Cys. Cell sheet shrunk and folded after the detachment from the redox responsive hydrogel (Scale bar is 200 µm).

**2-2.** **HepG2 spheroid formation using Elplasia System**

HepG2 spheroid cells were fabricated by using the 6-well plate Elpasia system that has 648 microholes. The initial HepG2 cells density was 2.4 x 10^4^ cells/mL or 150 cells/microhole. After 3 d culture, spheroids were harvested.

**
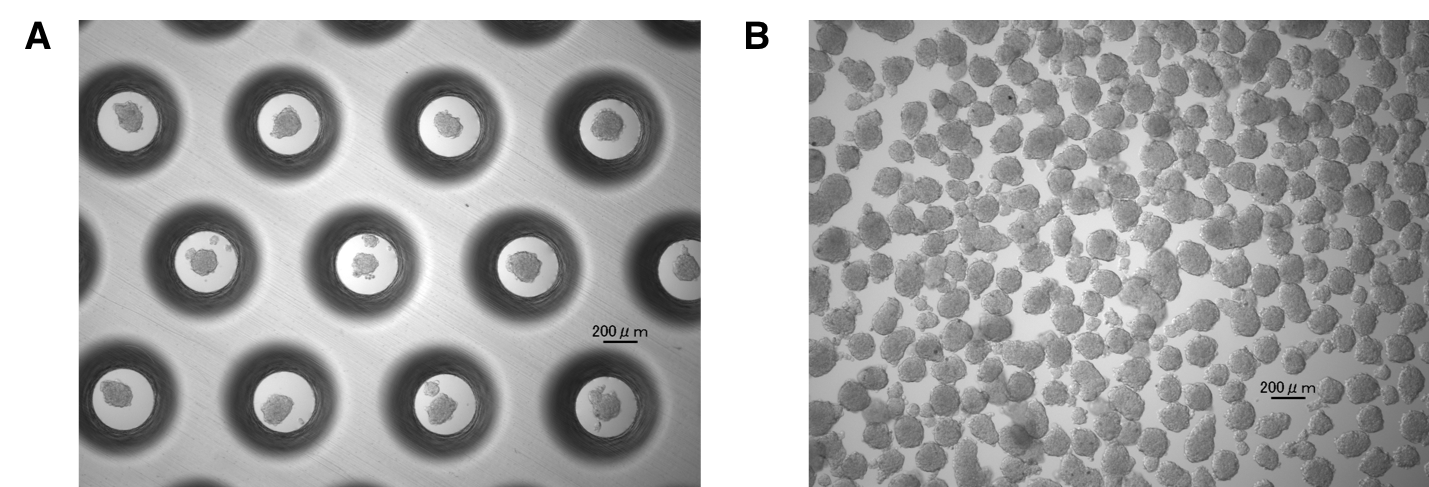
**

**Fig. S2**. Fabrication of HepG2 spheroid using Elplasia system. A. Seeding the HepG2 cells in the Elplasia™ microhole, B. Uniform HepG2 Spheroid formation after harvesting from Elplasia plate (Spheroid size is 119±21µm in diameter)

**2-3. Evaluation of closed structure of wrapped structure during co-culturing**

The prolonged incubation time until 7 d culturing has been conducted to evaluate the cellular distribution during the co-culture and assess the opened structure that showed in the one-day culture. Twenty-five of spheroids have been immobilized into the cell sheet surface and after HepG2 spheroids are completely layered by cell sheet, the wrapped structure was performed, and the images were captured by using a Keyence Microscope.


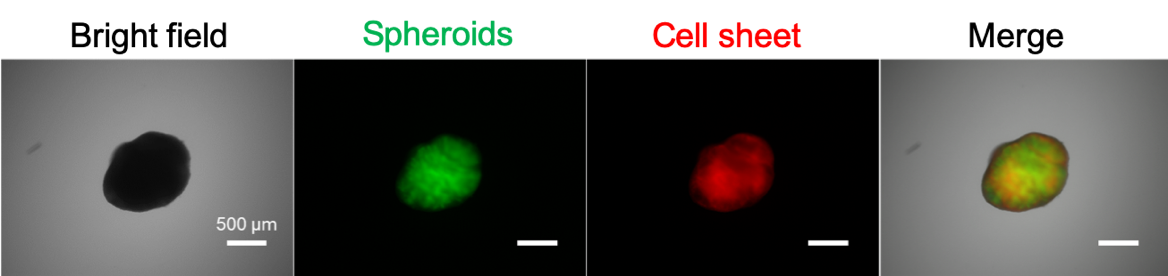


**Fig S3.** The closed position of wrapped structure after 7 d culturing. The NIH3T3 cell sheet is stained with DiD red fluorescence and HepG2 spheroids are stained with Calcein-AM green fluorescence.

- 1. **Evaluation of spheroid number in the wrapped structure**

To evaluate the spheroid number that can load into the wrapped structure, different number of HepG2 spheroids was seeded on the cell sheet surface. All hydrogel was degraded by using 20 mM of Cys. After 30 min incubation, wrapped structure were performed and the images were capture by using a Keyence Microscope.


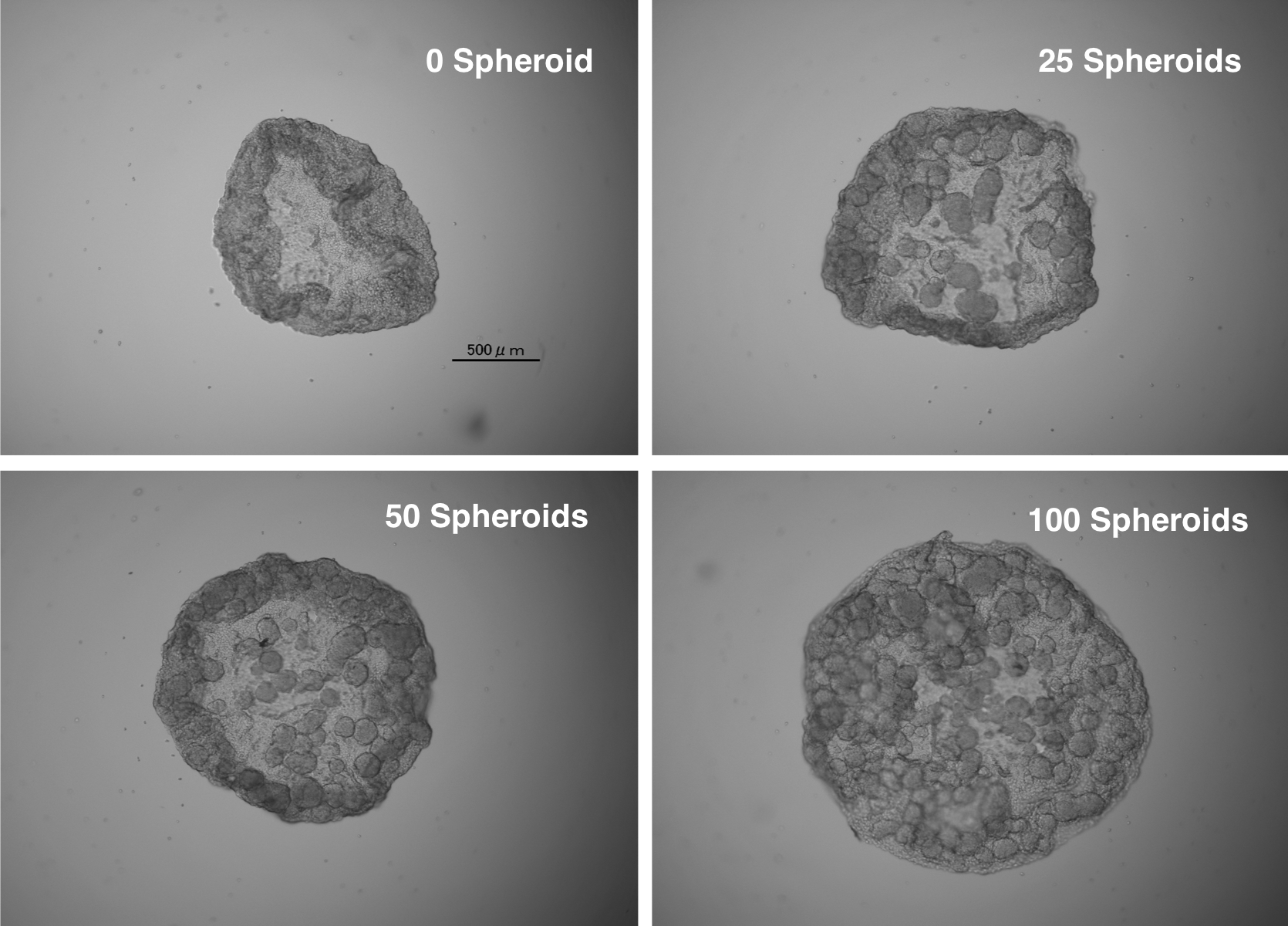


**Fig.S4** Various number of HepG2 spheroids in NIH3T3 cell sheet after the addition of Cys solution.

- 1. **Evaluation of collagen beads number in the wrapped structure**

First, the collagen beads or collagen microparticle was prepared by the membrane emulsification method^3^. Then, HUVECs (4000 cells per well) was seeded on the cell sheet with different collagen bead number (50, 150, 250 beads per well). All hydrogel was incubated for 4 h, then it was degraded by using 20 mM of Cys solution. After 30 min incubation, wrapped structure were performed and the images were capture by using a Keyence Microscope.


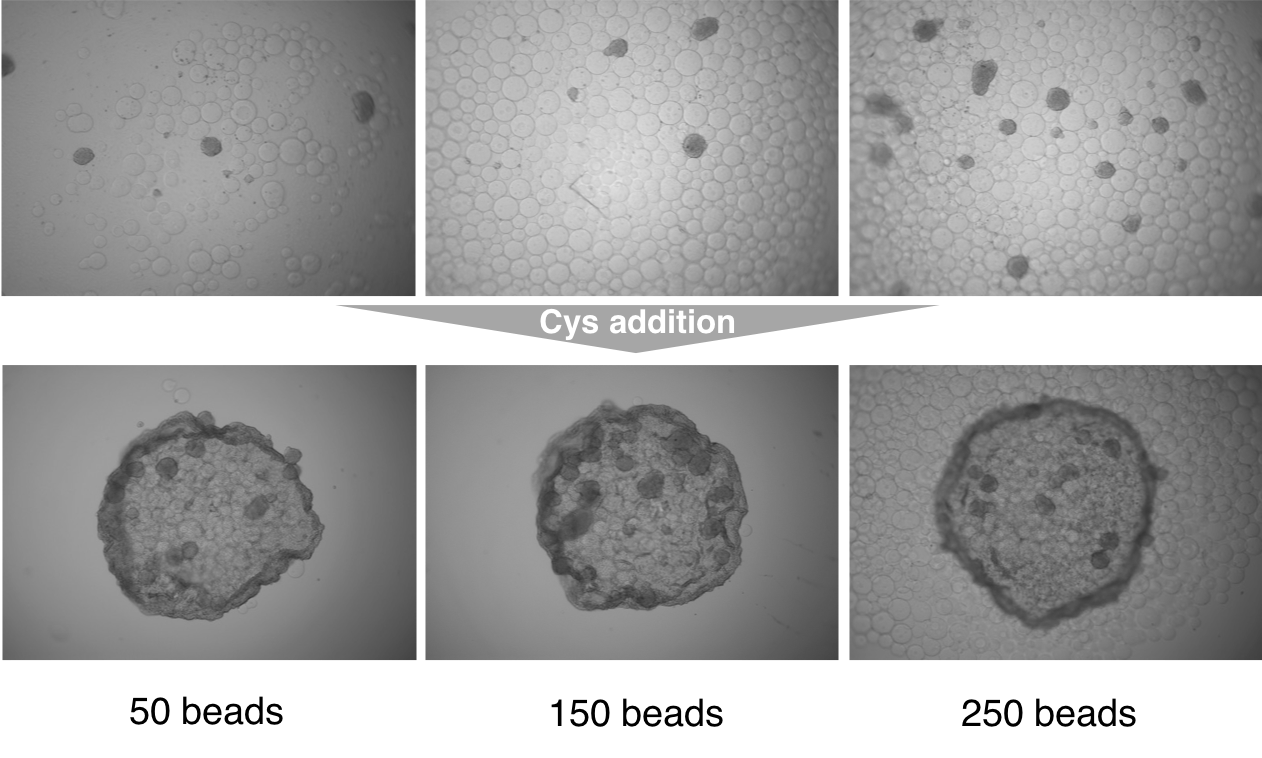


**Fig. S5.** Various number of collagen beads inside the wrapped structure.

**3. Supplementary Video Files**

**
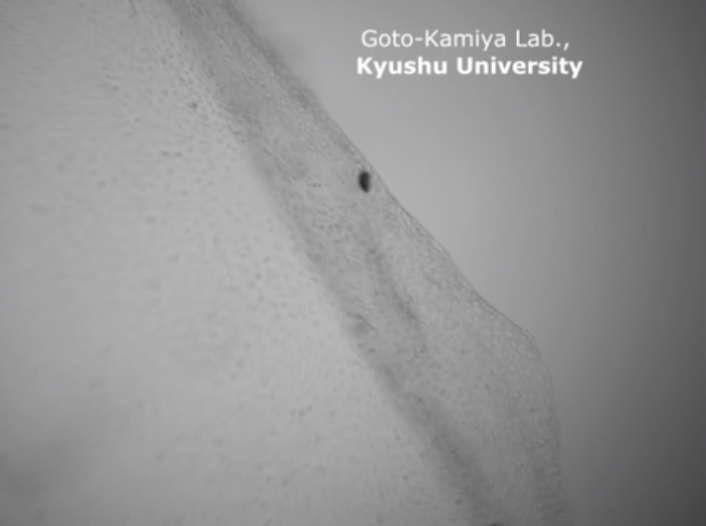
**

**Supplementary Video 1: Cell sheet detach from redox responsive hydrogel.**

The thumbnail image of cell sheet detachment from redox responsive hydrogel using 5 mM of Cys. Image shown the cell sheet is detached from hydrogel in mild condition, the folding occurs from the edge of cell sheet. Video is playing at 10x magnification in real-time capture of the detachment process of cell sheet.

**
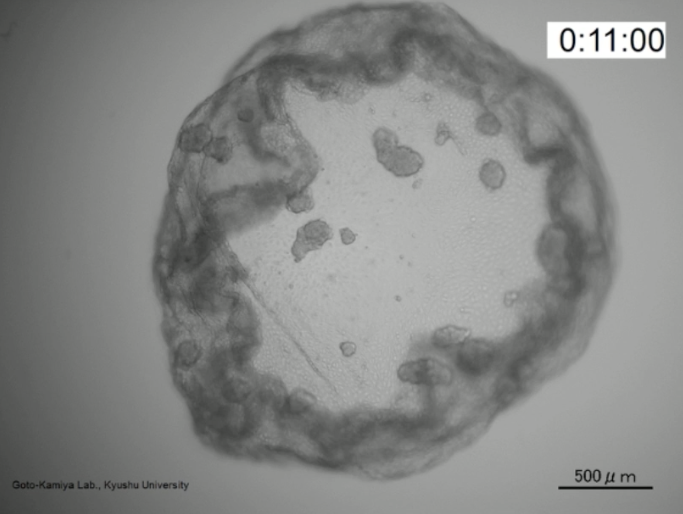
**

**Supplementary Video 2: Cellular Furoshiki: Cell sheet wrap the spheroids on redox responsive hydrogel**

The thumbnail image of cell sheet detachment from redox responsive hydrogel using 20 mM of Cys. Image shown the edge of cells started to fold around 9-11 min and all the HepG2 spheroids were carried to the centre of well over the wrapping process. Video is playing at 4x magnification in time-lapse capture of the detachment process of cell sheet.

**
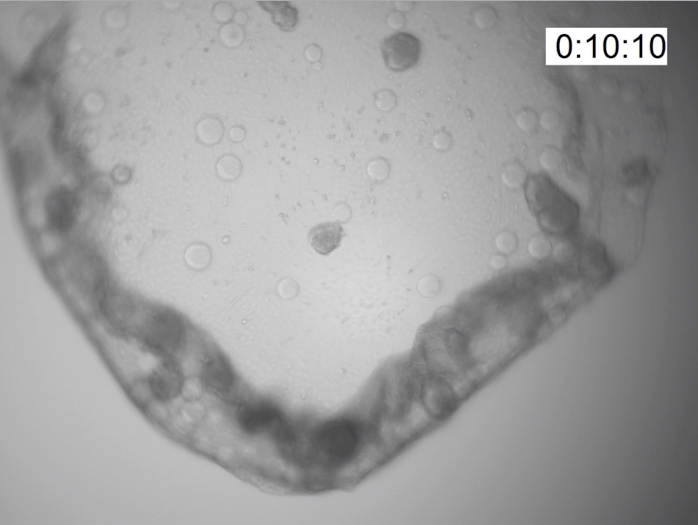
**

**Supplementary Video 3: Cellular Furoshiki with HepG2, Collagen beads, and HUVECs.**

The thumbnail image of cell sheet detachment from the redox responsive hydrogel using 20 mM of Cys as the reductant. Image shown the Cellular Furoshiki which consist with the co-cultured cells (15 spheroids/well of HepG2 and 4000 cells/well of HUVECs) and ca. 50 collagen beads. All the components were carried to the centre of well over the wrapping process. Video is playing at 4x magnification in time-lapse capture of the detachment process of cell sheet.

**4. Reference**

1. Moriyama, K., Wakabayashi, R., Goto, M. & Kamiya, N. Enzyme-mediated preparation of hydrogels composed of poly(ethylene glycol) and gelatin as cell culture platforms. *RSC Adv.* **5**, 3070–3073 (2015).

2. Ramadhan, W. *et al.* Enzymatically Prepared Dual Functionalized Hydrogels with Gelatin and Heparin To Facilitate Cellular Attachment and Proliferation. *ACS Appl. Bio Mater.* **2**, 2600–2609 (2019).

3. Yajima, Y., Yamada, M., Utoh, R. & Seki, M. Collagen Microparticle-Mediated 3D Cell Organization: A Facile Route to Bottom-up Engineering of Thick and Porous Tissues. *ACS Biomater. Sci. Eng.* **3**, 2144–2154 (2017).
